# Supplementary material for: Construction of Genetic Map and QTL Mapping for Seed Size and Quality Traits in Soybean (Glycine max L.)
Source: Int J Mol Sci. 2024 Mar 1;25(5):2857. doi: 10.3390/ijms25052857 (PMC10932342; doi:10.3390/ijms25052857)
Supplement: Supplementary file 1 [file ijms-25-02857-s001.zip › Figures S1-S2.pdf]

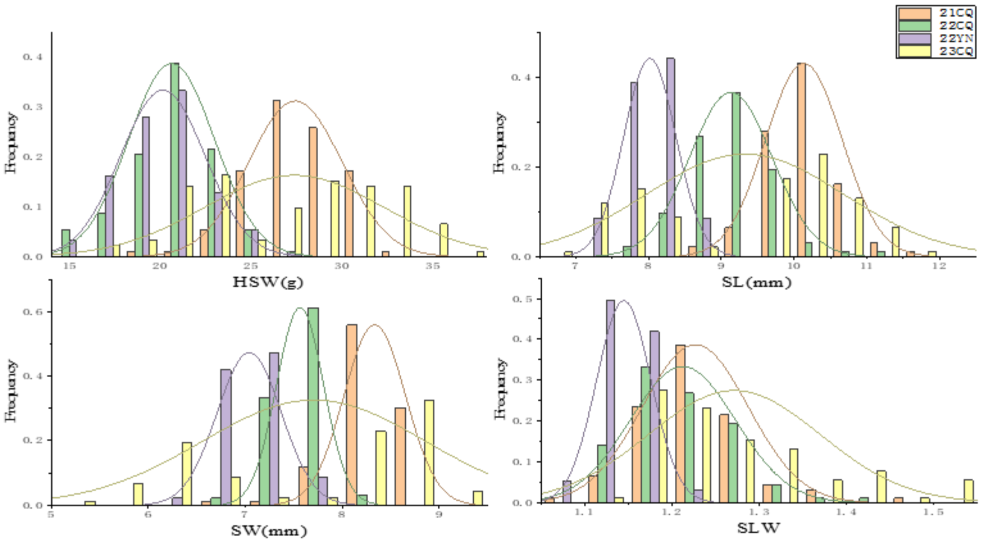

FIGURE S1 Frequency distributions of seed size traits in four environments.

HGW, hundred-grain weight (g); SL, seed length (mm);SW, seed width (mm); SLW, seed length-to-width ratio.

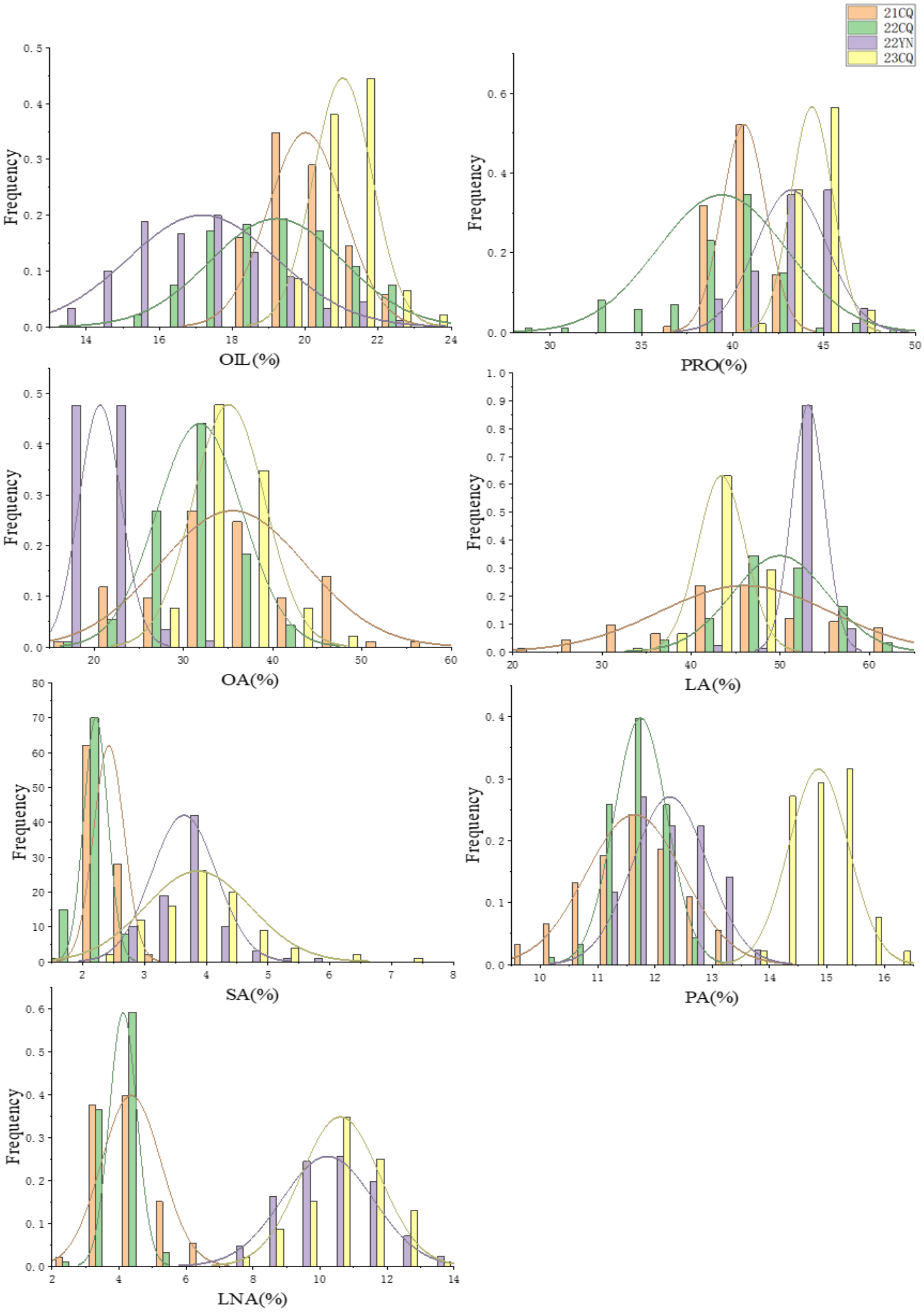

FIGURE S2 Frequency distributions of seed quality traits in four environments.

OIL: Oil content, PRO：Protein content, OA: oleic, LA: Linoleic acid, LNA: Linolenic acid, PA: palmitic acid, SA: stearic acid.
